# The Comparison of the Immunologic Properties of Stem Cells Isolated from Human Exfoliated Deciduous Teeth, Dental Pulp, and Dental Follicles

**DOI:** 10.1155/2016/4682875

**Published:** 2015-12-06

**Authors:** Selin Yildirim, Noushin Zibandeh, Deniz Genc, Elif Merve Ozcan, Kamil Goker, Tunc Akkoc

**Affiliations:** ^1^Division of Pediatric Allergy-Immunology, Faculty of Medicine, Marmara University, 34890 Istanbul, Turkey; ^2^Department of Oral and Maxillofacial Surgery, Faculty of Dentistry, Marmara University, 34890 Istanbul, Turkey

## Abstract

*Aim*. To compare the effects of various mesenchymal stem cells, those isolated from human exfoliated deciduous teeth (SHEDs), dental pulp stem cells (DPSCs), and dental follicle stem cells (DFSCs), on human peripheral blood mononuclear cells (PBMCs). *Method*. Mesenchymal stem cells were isolated from three sources in the orofacial region. Characterization and PCR analyses were performed. Lymphocytes were isolated from healthy peripheral venous blood. Lymphocytes were cocultured with stem cells in the presence and absence of IFN-*γ* and stimulated with anti-CD2, anti-CD3, and anti-CD28 for 3 days. Then, lymphocyte proliferation, the number of CD4^+^FoxP3^+^ T regulatory cells, and the levels of Fas/Fas ligand, IL-4, IL-10, and IFN-*γ* in the culture supernatant were measured. *Results*. The DFSCs exhibited an enhanced differentiation capacity and an increased number of CD4^+^FoxP3^+^ T lymphocytes and suppressed the proliferation and apoptosis of PBMCs compared with SHEDs and DPSCs. The addition of IFN-*γ* augmented the proliferation of DFSCs. Furthermore, the DFSCs suppressed IL-4 and IFN-*γ* cytokine levels and enhanced IL-10 levels compared with the other cell sources. *Conclusion*. These results suggest that IFN-*γ* stimulates DFSCs by inducing an immunomodulatory effect on the PBMCs of healthy donors while suppressing apoptosis and proliferation and increasing the number of CD4^+^FoxP3^+^ cells.

## 1. Introduction

Stem cells possess self-renewal capacity and are able to differentiate into various types of cells in the body. Hence, they induce the repair and regeneration of tissues and organs, making stem cells an ideal source for cell therapy and regenerative medicine. Stem cells are primarily divided into two groups: embryonic stem cells and adult stem cells. Embryonic stem cells have pluripotent characteristics and can differentiate into most embryonic layers [1]. Due to ethical issues that surround the use of embryonic stem cells, most recent studies have focused on adult-derived stem cells.

Mesenchymal stem cells (MSCs) are multipotent adult stem cells. MSC populations have been isolated from various sources, such as cord blood [2, 3], Wharton's jelly [4], the placenta [5, 6], bone marrow [7], teeth [8], and adipose tissue [9, 10].

A promising source of MSCs is dental tissue, which is easily accessible and can be isolated from many sources of the orofacial region, such as stem cells isolated from human exfoliated deciduous teeth (SHEDs) [8], dental pulp stem cells (DPSCs) [11], dental follicle stem cells (DFSCs) [12], and periodontal ligament stem cells (PDLSCs) [13]. These MSCs (SHED, DPSCs, and DFSCs) are able to differentiate into osteoblasts, adipocytes, and chondrocytes under suitable conditions. These cells also express MSC-specific markers, such as CD29, CD73, CD90, CD105, and CD166, and are negative for hematopoietic markers, including CD14, CD45, CD34, CD25, and CD28 [8, 11–13].

The immune system is a major defense mechanism that provides protection against foreign substances and produces a variety of cells and molecules that can recognize and eliminate the vast variety of possible foreign materials. The first protective barrier against microorganisms is the natural (innate) immune response [14]. Regulatory T (Treg) cells play an important role in controlling immune responses to allergens, autoantigen tumor antigens, and infectious agents. These cells express the transcription factor fork head box P3 (FoxP3) [15, 16].

Recent studies on MSCs have reported their inhibitory effect on lymphocyte proliferation [17, 18]. Later, adaptive immune responses develop for specific pathogens. MSCs have immunosuppressive and immunomodulatory effects that are promising for the treatment of autoimmune diseases. MSCs suppress mitogen-stimulated memory and naive T cell responses. The suppressive effect of MSCs increases T cell viability and decreases the related cell apoptosis [19].

Most studies on T cells have reported mesenchymal stem-immunosuppressive cell interactions. T cells are the primary cellular effectors of the adaptive immune system and play a fundamental role in cell-mediated immunity. A number of reports have shown that the anti-inflammatory and immunomodulatory effects of MSCs are associated with the inhibition of T cell proliferation and cytokine production by effector T cell subsets.

MSCs affect the various types of T-helper cell subtypes (Th1, Th2, and Th17 cells) and Treg cells via various mechanisms. Human MSCs downregulate interferon-gamma expression via T-helper type 1, upregulate interleukin-4 expression via T-helper type 2 cells, increase the ratio of Treg cells, and cause shift from a proinflammatory environment towards an anti-inflammatory environment. The mechanisms that mediate the inhibitory effects of MSCs are not yet clearly defined [20]. Cell-cell contact and soluble factors are believed to induce suppressive effects. SHED cells were found to have a significant effect on Th17 cell inhibition compared with bone marrow mesenchymal stem cells (BMMSCs) [21].

In this study, we investigated the immunomodulatory effects of SHEDs, DPSCs, and DFSCs on the peripheral blood mononuclear cells (PBMCs) of healthy donors. Thus, coculturing of SHEDs, DPSCs, and DFSCs with PBMCs that were isolated from venous blood samples affected the immune system cells, indicating that stem cells play a primary role in the mechanisms of the immune system.

## 2. Materials and Methods

### 2.1. Isolation of Stem Cells

Dental pulp (DP), dental follicle (DF), and SHED cells were collected from the Marmara University Faculty of Dentistry Oral and Maxillofacial Surgery. The legitimate delegate of all patients provided informed consent according to the guidelines of the Ethics Committee of the Marmara University Medical Faculty in Istanbul, Turkey (09.2014.0015/70737436-050.06.04). Teeth were obtained from 3 children aged from 8 to 15 years and 3 adult donors aged 20–25 years undergoing third-molar extraction, and healthy human third-molar follicles were collected from the tooth buds of 3 healthy teeth aged 20–25 years. These pulps and follicles were transported in Dulbecco's phosphate-buffered saline (DPBS, Gibco, Grand Island, NY 14072, USA) containing 1% penicillin/streptomycin (Gibco, USA). All laboratory work was performed in a laboratory in the Department of Pediatric Allergy-Immunology, Marmara University Research Hospital.

Dental pulps and follicles were isolated under sterile conditions. Pulps and follicles were enzymatically treated with 3 mg/mL collagenase type I (Gibco, USA) for 45 minutes at 37°C to completely digest pulps and follicles tissue. Then, 3 mL of Dulbecco's modified Eagle's medium (DMEM, Gibco, USA) supplemented with 10% fetal bovine serum (FBS, Gibco, USA) and 1% penicillin/streptomycin was added to digest the pulp and follicle tissue followed by centrifugation at 1200 rpm for 5 minutes. Cells pellets were obtained, and the supernatant was aspirated. DPSCs, SHEDs, and DFSCs were cultivated in T-25 flasks in a 5% CO_2_ atmosphere under 37°C in culture medium composed of DMEM, 10% FBS, and 1% penicillin/streptomycin. The stem cells were washed with DPBS and provided with fresh culture medium. The culture medium was changed every 3 to 4 days until the cells reached confluence. The cells were detached with 0.25% trypsin-EDTA (Gibco, USA) when they reached 70–80% confluence. Adherent cells cultured for 3 passages were characterized and analyzed for specific surface markers. The cellular analyses and differentiation were performed using flow cytometry.

### 2.2. Flow Cytometry Analysis

To analyze the cell surface antigen expressions, the cells from the third passage were used. SHED, DFSCs, and DPSCs were incubated with antibodies for human CD73 phycoerythrin (PE), CD90 PE, CD146 fluorescein isothiocyanate (FITC), CD29 allophycocyanin (APC), CD105 PE, CD45 FITC, CD34 PE, CD14 PE, CD25 APC, and CD28 PE (BD Biosciences, San Diego, CA, USA) at room temperature in the dark. Control antibodies were phycoerythrin-conjugated or fluorescein isothiocyanate-conjugated and allophycocyanin-conjugated mouse IgG1 and mouse IgG_2_ (BD Biosciences, San Diego, CA, USA). The flow cytometry results were analyzed using BD FACS Calibur.

### 2.3. Differentiation of Stem Cells

To induce osteogenic (MesenCult, Stemcell Technologies, North America), adipogenic, and chondrogenic differentiation, a human MSC functional identification kit (Gibco, Grand Island, USA) was used. For differentiation, the cells were plated in 6-well plates (5 × 10^4^ cell/well), and the differentiation medium was prepared according to the manufacturer's instructions and changed 3 times per week. After 14 days, the adipocytes and chondrocytes were stained with Oil Red O and Alcian blue, respectively, and after 28 days, the osteocytes were stained with Alizarin red.

### 2.4. Real-Time PCR Analysis

Total RNA was isolated from 10 × 10^6^ SHEDs, DPSCs, and DFSCs at passage 3 using a high pure RNA isolation kit (Roche, Mannheim, Germany) according to the manufacturer's instructions. One microgram of total RNA was converted to cDNA using a transcriptor first strand cDNA synthesis kit (Roche, Mannheim, Germany). Equal amounts of cDNA were used for the real-time amplification of the target genes according to the manufacturer's recommendations using a LightCycler 480 Real-Time PCR System (Roche Diagnostic, Mannheim, Germany). The gene expression of specific markers for MSCs, including ALPL, RUNX2, NANOG, NESTIN, NOTCH, and DSPP, was quantified relative to the housekeeping gene GAPDH. The RT-PCR conditions were as follows: preincubation for 10 minutes at 95°C for 1 cycle; amplification for 10 seconds at 95°C, 60°C for 30 seconds, and 72°C for 1 second for 45 cycles; and cooling for 10 seconds at 40°C for 1 cycle. The reaction mixture lacking cDNA was used as a negative control in each run. The real-time PCR results were analyzed using LightCycler software (version 2).

### 2.5. Lymphocyte Isolation

Peripheral blood was obtained from 10 healthy donors aged from 20 to 25 years and was added to heparin tubes. The legitimate delegate of all patients provided informed consent according to guidelines of the Ethical Committee of the Marmara University Medical Faculty in Istanbul, Turkey. PBMCs were obtained via Ficoll-Paque (GE Healthcare Bio-Sciences) density gradient from heparinized peripheral blood samples, as previously described [22]. The cells were cultured in RPMI (Gibco, USA) supplemented with 10% FBS and 1% penicillin/streptomycin. PBMCs were stimulated with 5 *μ*L of anti-CD2 (0.5 *μ*g/mL, eBioscience, San Diego, CA)/anti-CD3 (0.5 *μ*g/mL, Life Span Biosciences, USA)/anti-CD28 (0.5 *μ*g/mL, Millipore, California) (CDmix) at 37°C in a humidified atmosphere containing 5% CO_2_ for 72 h.

### 2.6. Coculture of Human PBMCs with SHEDs, DPSCs, and DFSCs

SHEDs, DPSCs, and DFSCs (5 × 10^4^/well in a 48-well plate) were plated 48 h prior to the addition of ten times number of lymphocytes in the culture medium. SHEDs, DPSCs, DFSCs, and lymphocytes (1 : 10) were cocultured for 3 days. The cultures were stimulated using 5 *μ*L of the CDmix and stimulated with and without 5 *μ*L of IFN-*γ* (5 *μ*g/mL, Millipore, CA, USA). Then, lymphocyte proliferation (carboxyfluorescein succinimidyl ester, CFSE), apoptosis (Fas/Fas ligand), CD4^+^FoxP3^+^ Treg cell expression and cytokine expression were analyzed via flow cytometry.

### 2.7. CFSE Assays for Lymphocyte Proliferation

After 3 days of coculturing, the cell proliferation behavior of the lymphocytes was quantified using carboxyfluorescein succinimidyl ester (CFSE) (Invitrogen, Grand Island, USA). The cells were labeled with CFSE, and 10 *μ*m CFSE dye was used to stain the lymphocytes after coculturing. The lymphocytes were stimulated in vitro with and without SHEDs, DPSCs, and DFSCs and were tested for CFSE dilution via flow cytometry.

### 2.8. Detection of Apoptosis of the Lymphocytes by Fas/Fas Ligand Labeling

After 3 days of coculturing, the apoptotic rate of the lymphocytes was quantified using a Fas/Fas ligand kit (BD Biosciences, USA), according to the manufacturer's instructions. The kit included CD95-FITC, CD178-biotin, IgG1 *κ* isotype control-biotin, and PE streptavidin.

### 2.9. CD4^+^FoxP3^+^ Treg Cell Assessment

After 3 days of coculturing, the Treg lymphocyte cells were quantified using a Human FoxP3 Buffer Set (BD Biosciences, USA). We determined the percentage of Treg (CD4^+^FoxP3^+^) markers that had developed from the lymphocytes. The cultures were assessed via flow cytometry using Human FoxP3 Buffer Kit according to the manufacturer's instructions. The kit included Buffer A, and anti-human CD4 and anti-human FoxP3 PE (BD Biosciences, USA).

### 2.10. Analysis of the Cytokine Expression Profiles

After 3 days of coculture, the supernatant percentages of IL-10, IL-4, and IFN-*γ* were measured. The cytokines were analyzed via flow cytometry using a BD cytometric bead array (CBA) human Th1/Th2/Th17 Kit (BD Biosciences, USA) according to the manufacturer's instructions.

### 2.11. Statistical Analyses

The differences between groups were analyzed via a one-way ANOVA test using SPSS v20 (SPSS Inc., Chicago, IL, USA) and GraphPad Prism 6 software. Graphs were generated using GraphPad Prism. *P* values less than 0.05 were considered significant.

## 3. Results

### 3.1. Isolation, Characterization, Differentiation, and Real-Time PCR Analysis of SHEDs, DPSCs, and DFSCs

**Figure 1:**
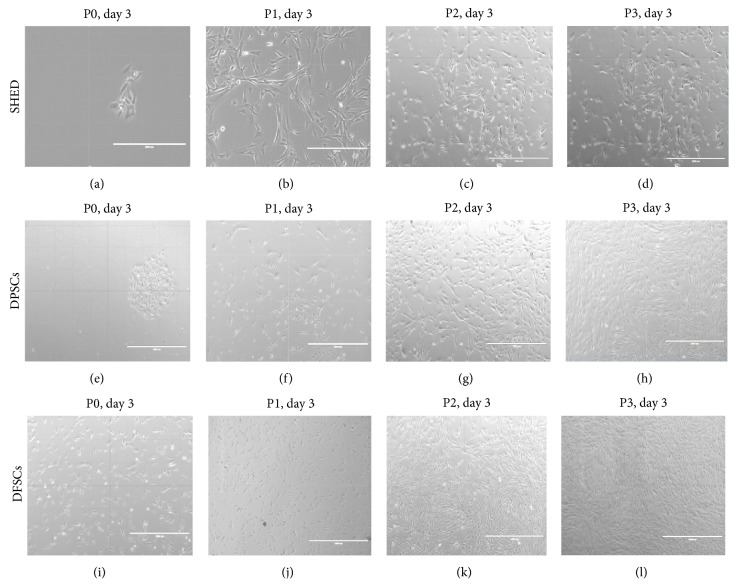
Morphological appearance of SHED, DPSCs, and DFSCs. Morphology of SHED, (a) P0: 3rd day, (b) P1: 3rd day, (c) P2: 3rd day, and (d) P3: 3rd day. Original magnifications: (a and b) ×20; (c and d) ×10. Morphology of DPSCs, (e) P0: 3rd day, (f) P1: 3rd day, (g) P2: 3rd day, and (h) P3: 3rd day. Original magnifications: (e, f, g, and h) ×10. Morphology of DFSCs, (i) P0: 3rd day, (j) P1: 3rd day, (k) P2: 3rd day, and (l) P3: 3rd day. Original magnifications: (i) ×20; (j, k, and l) ×10.

SHEDs, DPSCs, and DFSCs attached sparsely to the culture flasks and exhibited a fibroblast-like and spindle-shaped morphology during the early days of incubation. The SHEDs began to proliferate in approximately 3 days and gradually formed small colonies (Figure 1(a)). The SHEDs reached 70% confluency in the primary culture 7 days after being plated in their first passage (P1). Most of the SHEDs exhibited fibroblast-like morphology in the later passages (P1, P2, and P3; Figures 1(b)–1(d)). The DPSCs began to proliferate in approximately 4-5 days and gradually formed small colonies (Figure 1(e)). The DPSCs reached 70% confluency in the primary culture 9 days after being plated in their first passage (P1). Most of the DPSCs exhibited a fibroblast-like morphology in the later passages (P1, P2, and P3; Figures 1(f)–1(h)). The DFSCs began to proliferate in approximately 2 days and gradually formed small colonies (Figure 1(a)). The DFSCs reached 70% confluency in the primary culture 5-6 days after being plated in their first passages (P1). Most of the DFSCs exhibited fibroblast-like morphology in the later passages (P1, P2, and P3; Figures 1(j)–1(l)). Then, immunophenotyping and differentiation of the three cell passages were observed.

**Figure 2:**
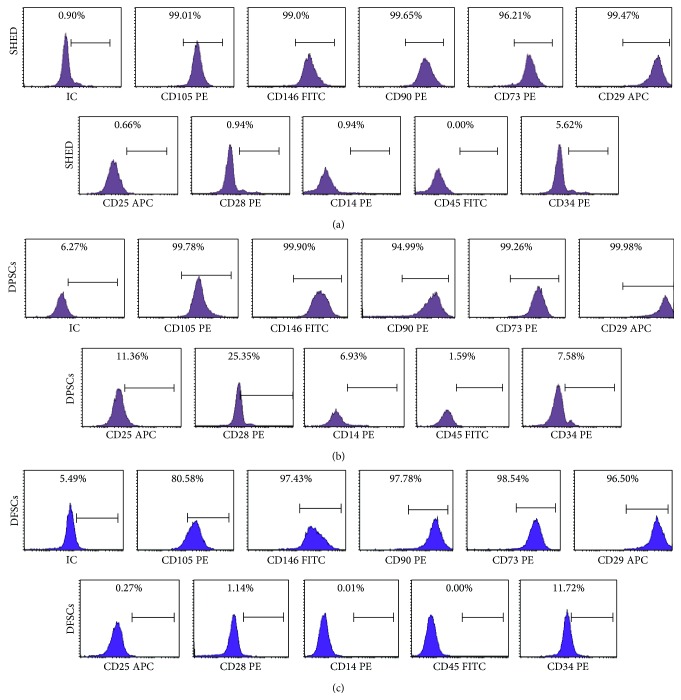
Representative flow cytometry analysis of cell surface markers in SHED, DPSCs, and DFSCs. Representative flow cytometry analysis of cell surface markers (a) on SHED in P3, (b) on DPSCs in P3, and (c) on DFSCs in P3.

The SHEDs, DPSCs, and DFSCs were analyzed via flow cytometry. These cells exhibited positive staining for CD29, CD73, CD90, CD105, and CD146 but were negative for CD14, CD25, CD28, CD34, and CD45 (Figures 2(a)–2(c)).

**Figure 3:**
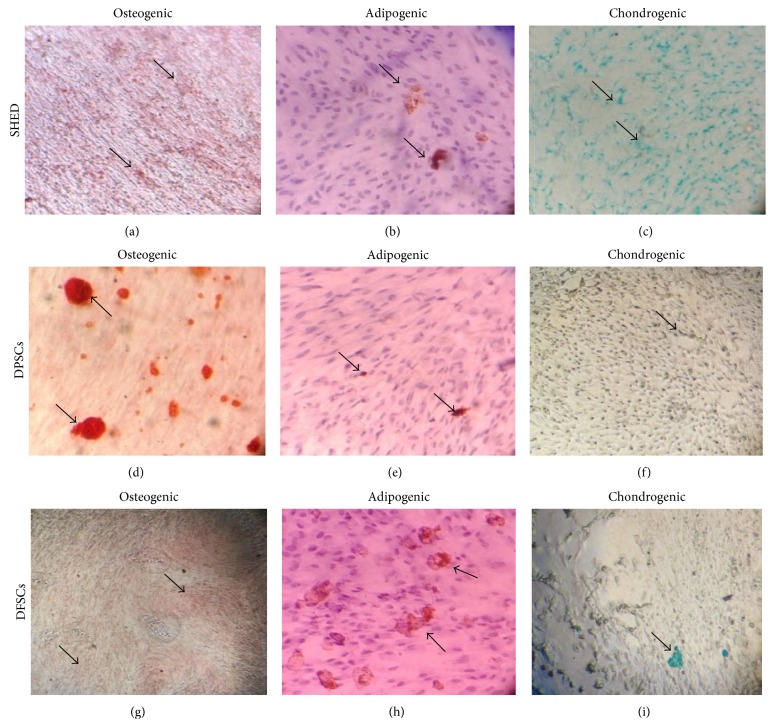
Differentiation analysis in SHED, DPSCs, and DFSCs. (a, d, g) Alizarin red staining of osteogenic induced SHED, DPSCs, and DFSCs. (b, e, h) Oil Red staining of adipogenic induced SHED, DPSCs, and DFSCs. (c, f, i) Alcian blue staining of chondrogenic induced SHED, DPSCs, and DFSCs.

The SHEDs, DPSCs, and DFSCs differentiated into osteocytes, adipocytes, and chondrocytes. First, the osteogenic differentiation capability was investigated in vitro during a twenty-eight-day culture period in osteogenic induction medium. The SHEDs, DPSCs, and DFSCs were stained with Alizarin red, and the cells formed calcified bone nodule structures (Figures 3(a), 3(d), and 3(g)). Next, the in vitro adipogenic differentiation capability was assessed by culturing the cells in adipogenic induction medium and staining with Oil Red O. Intracellular lipid droplets were observed in these cells (Figures 3(b), 3(e), and 3(h)). Finally, the chondrogenic differentiation capability was investigated in vitro during a fourteen-day culture period in chondrogenic induction medium, and cell differentiation into chondrocytes was confirmed with Alcian blue staining. Intracellular proteoglycans were observed in these cells (Figures 3(c), 3(f), and 3(i)).

**Figure 4:**
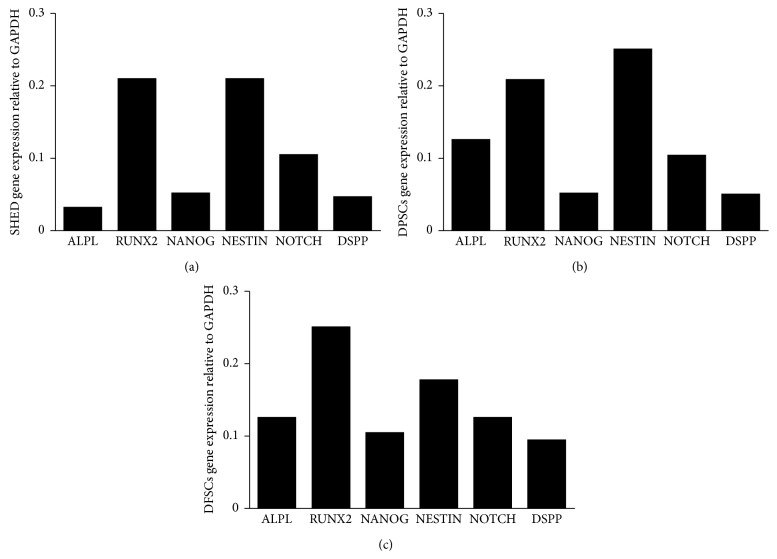
Gene expression of specific markers for SHED (a), DPSCs (b), and DFSCs (c), including ALPL (alkaline phosphatase), RUNX2 (runt-related transcription factor 2), NANOG, NESTIN, NOTCH, and DSPP (dentin sialophosphoprotein) according to housekeeping gene GAPDH (glyceraldehyde 3-phosphate dehydrogenase) was performed.

We analyzed the gene expression of specific markers in SHEDs, DPSCs, and DFSCs, including alkaline phosphatase (ALP), runt-related transcription factor 2 (RUNX2), NANOG, NESTIN, NOTCH, and dentin sialophosphoprotein (DSPP) relative to the housekeeping gene, glyceraldehyde 3-phosphate dehydrogenase (GAPDH). The SHEDs, DPSCs, and DFSCs expressed ALPL, RUNX2, NANOG, NESTIN, NOTCH, and DSPP genes. The DFSCs expressed higher levels of all genes compared with SHEDs and DPSCs (Figures 4(a)–4(c)).

### 3.2. The DFSCs Suppressed Lymphocyte Proliferation Better Than the SHED Cells and DPSCs

**Figure 5:**
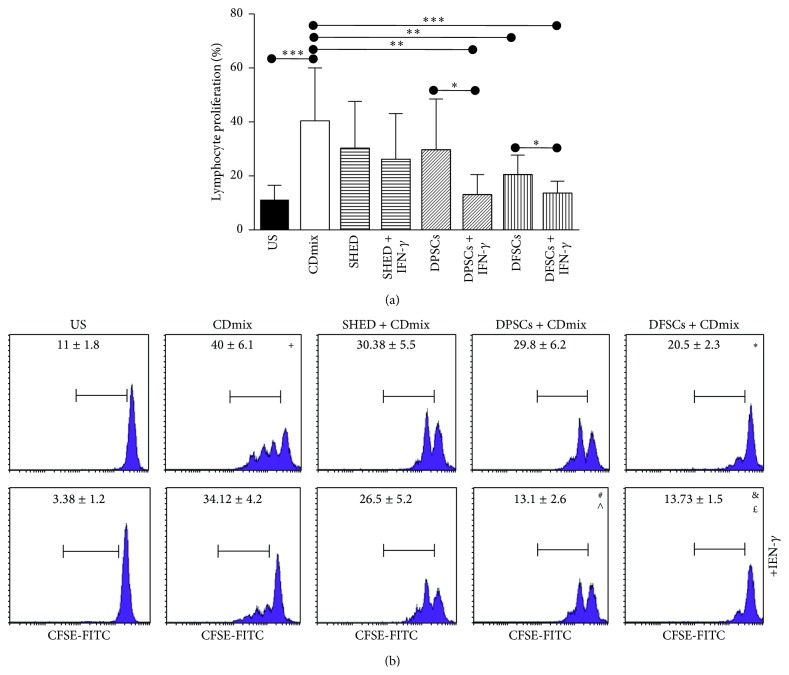
Inhibitory effect of SHED, DPSCs, and DFSCs on the proliferation of lymphocytes as detected by CFSE. (a) Inhibitory effect of SHED, DPSCs, and DFSCs on the proliferation of lymphocytes displayed statistically. (b) Inhibitory effect of SHED, DPSCs, and DFSCs on the proliferation of lymphocytes displayed by flow cytometry. ^+^ *P* < 0.001, compared with US group. ^*∗*^ *P* < 0.01, compared with CDmix group. ^#^ *P* < 0.05, compared with DPSCs + CDmix group. ^&^ *P* < 0.05, compared with DFSCs + CDmix group. ^∧^ *P* < 0.01, compared with CDmix group. ^£^ *P* < 0.001, compared with CDmix group.

Lymphocyte proliferation was quantified via flow cytometry. In the CFSE labeling assay, lymphocyte proliferation was suppressed at day 3. The proliferation of lymphocytes stimulated with the CDmix was significantly increased compared with unstimulated lymphocytes (*P* < 0.001). The proliferation of the lymphocytes stimulated with the CDmix in the presence and absence of IFN-*γ* was suppressed when the lymphocytes were cocultured with SHED cells, although the result was not significant (*P* > 0.05). The proliferation of the lymphocytes stimulated with the CDmix was suppressed when the lymphocytes were cocultured with DPSCs, although the result was not significant (*P* > 0.05). However, the proliferation of the lymphocytes stimulated with the CDmix in the presence of IFN-*γ* was significantly suppressed (*P* < 0.01). The proliferation of the lymphocytes stimulated with the CDmix in the presence of IFN-*γ* was significantly suppressed (*P* < 0.05) compared with the proliferation of the lymphocytes in the absence of IFN-*γ*. The proliferation of the lymphocytes stimulated with the CDmix was significantly suppressed when the lymphocytes were cocultured with DFSCs (*P* < 0.01). In addition, the proliferation of the lymphocytes stimulated with the CDmix in the presence of IFN-*γ* was significantly suppressed (*P* < 0.001), and the proliferation of the lymphocytes was significantly suppressed (*P* < 0.05) when stimulated with the CDmix in the presence of IFN-*γ* compared with in the absence of IFN-*γ* (Figures 5(a) and 5(b)).

### 3.3. The DFSCs Suppressed Apoptotic Effects Better Than SHED Cells and DPSCs

**Figure 6:**
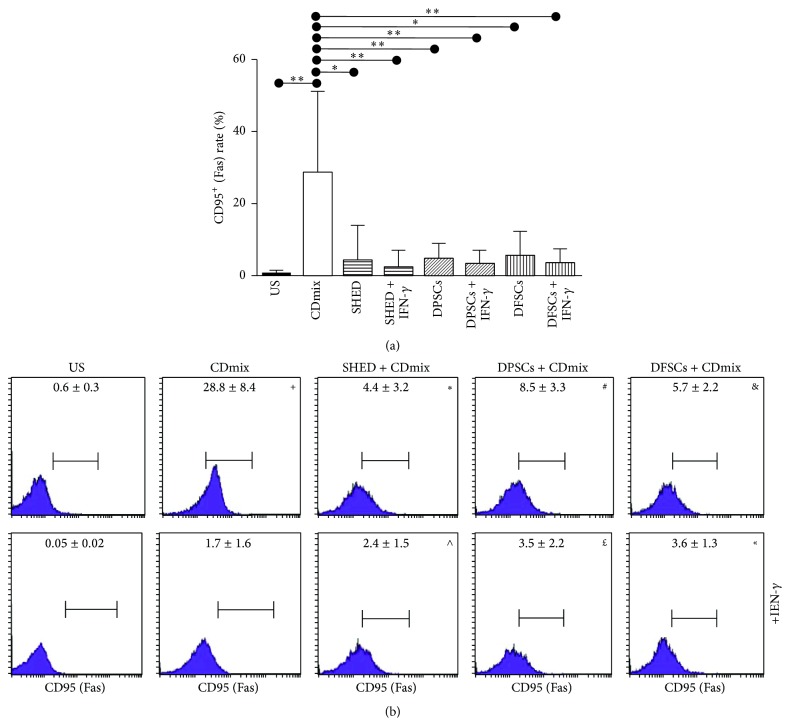
Inhibitory effect of SHED, DPSCs, and DFSCs on the apoptosis of lymphocytes as detected by Fas/FasLigand kit. (a) Inhibitory effect of SHED, DPSCs, and DFSCs on Fas (CD95) rate of lymphocytes displayed statistically. (b) Inhibitory effect of SHED, DPSCs, and DFSCs on Fas (CD95) rate of lymphocytes displayed by flow cytometry. ^+^ *P* < 0.01, compared with US group. ^*∗*^ *P* < 0.05, compared with CDmix group. ^#^ *P* < 0.01, compared with CDmix group. ^&^ *P* < 0.05, compared with CDmix group. ^∧^ *P* < 0.01, compared with CDmix group. ^£^ *P* < 0.01, compared with CDmix group. ^«^ *P* < 0.01, compared with CDmix group.

The Fas/Fas ligand rates of the lymphocytes were quantified via flow cytometry. The inhibitory effect of SHEDs, DPSCs, and DFSCs on the Fas (CD95) rate of the lymphocytes was significant. The Fas (CD95) rate of the lymphocytes stimulated with the CDmix was significantly increased (*P* < 0.01) compared with unstimulated lymphocytes. The Fas (CD95) rate of the lymphocytes stimulated with the CDmix was significantly suppressed when the lymphocytes were cocultured with SHED cells (*P* < 0.05). In addition, the Fas (CD95) rate of the lymphocytes stimulated with the CDmix in the presence of IFN-*γ* was significantly suppressed (*P* < 0.01). The Fas (CD95) rate of the lymphocytes stimulated with the CDmix was significantly suppressed when the lymphocytes were cocultured with the DPSCs (*P* < 0.01). In addition, the Fas (CD95) rate of the lymphocytes stimulated with the CDmix in the presence of IFN-*γ* was significantly suppressed (*P* < 0.01). The Fas (CD95) rate of the lymphocytes stimulated with the CDmix was significantly suppressed when the lymphocytes were cocultured with the DFSCs (*P* < 0.05). Finally, the Fas (CD95) rate of the lymphocytes stimulated with the CDmix in the presence of IFN-*γ* was significantly suppressed (*P* < 0.01; Figures 6(a) and 6(b)).

**Figure 7:**
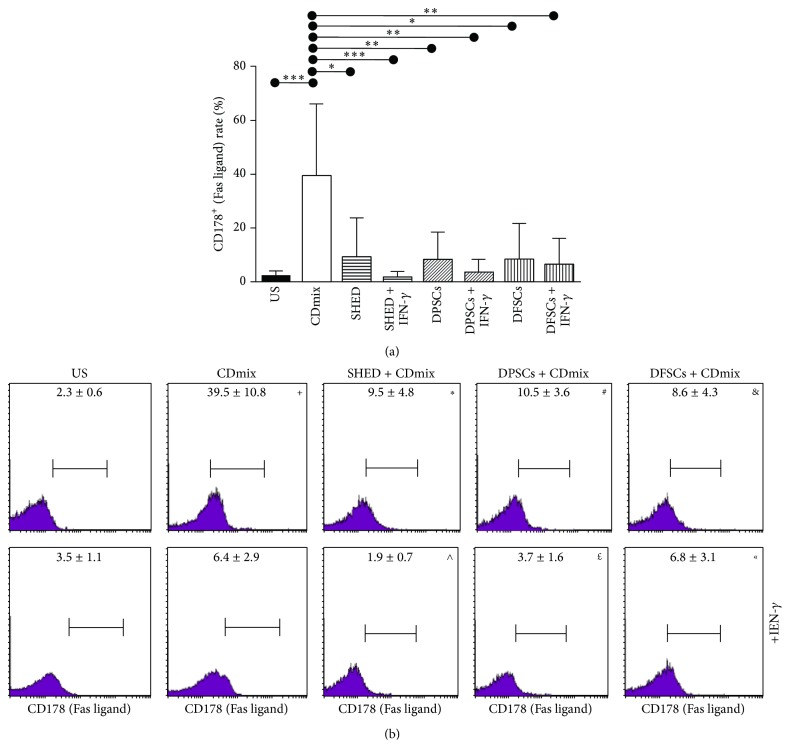
Inhibitory effect of SHED, DPSCs, and DFSCs on the apoptosis of lymphocytes as detected by Fas/FasLigand kit. (a) Inhibitory effect of SHED, DPSCs, and DFSCs on Fas ligand (CD178) rate of lymphocytes displayed statistically. (b) Inhibitory effect of SHED, DPSCs, and DFSCs on Fas ligand (CD178) rate of lymphocytes displayed by flow cytometry. ^+^ *P* < 0.001, compared with US group. ^*∗*^ *P* < 0.05, compared with CDmix group. ^#^ *P* < 0.01, compared with CDmix group. ^&^ *P* < 0.05, compared with CDmix group. ^∧^ *P* < 0.001, compared with CDmix group. ^£^ *P* < 0.01, compared with CDmix group. ^«^ *P* < 0.01, compared with CDmix group.

The inhibitory effect of SHEDs, DPSCs, and DFSCs on the Fas ligand (CD178) rate of the lymphocytes was significant. The Fas ligand (CD178) rate of the lymphocytes stimulated with the CDmix was significantly increased (*P* < 0.001) compared with the unstimulated lymphocytes. The Fas ligand (CD178) rate of the lymphocytes stimulated with the CDmix was significantly suppressed when the lymphocytes were cocultured with SHED cells (*P* < 0.05). In addition, the Fas ligand (CD178) rate of the lymphocytes stimulated with the CDmix in the presence of IFN-*γ* was significantly suppressed (*P* < 0.001). The Fas ligand (CD178) rate of the lymphocytes stimulated with the CDmix was significantly suppressed when the lymphocytes were cocultured with the DPSCs (*P* < 0.01). In addition, the Fas ligand (CD178) rate of the lymphocytes stimulated with the CDmix in the presence of IFN-*γ* was significantly suppressed (*P* < 0.01). The Fas ligand (CD178) rate of the lymphocytes stimulated with the CDmix was significantly suppressed when the lymphocytes were cocultured with the DFSCs (*P* < 0.05). In addition, the Fas ligand (CD178) rate of the lymphocytes stimulated with the CDmix in the presence of IFN-*γ* was significantly suppressed (*P* < 0.01; Figures 7(a) and 7(b)).

### 3.4. Effects of SHEDs, DPSCs, and DFSCs on CD4^+^FoxP3^+^ Treg Cell Expansion of Lymphocytes

**Figure 8:**
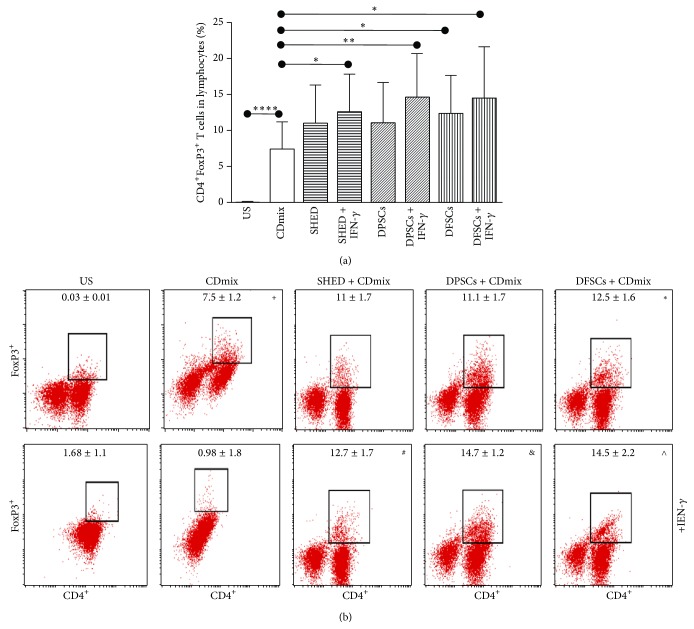
We therefore studied the effect of SHED, DPSCs, and DFSCs on Treg frequency. (a) Induced effects of SHED, DPSCs, and DFSCs on CD4^+^FoxP3^+^ Treg cells were displayed statistically. (b) Induced effects of SHED, DPSCs, and DFSCs on CD4^+^FoxP3^+^ Treg cells were displayed by flow cytometry. ^+^ *P* < 0.0001, compared with US group. ^*∗*^ *P* < 0.05, compared with CDmix group. ^#^ *P* < 0.05, compared with CDmix group. ^&^ *P* < 0.01, compared with CDmix group. ^∧^ *P* < 0.05, compared with CDmix group.

We studied the effects of SHEDs, DPSCs, and DFSCs on the Treg frequency. CD4^+^FoxP3^+^ Treg cells were significantly induced by the stimulated lymphocytes with the CDmix compared with unstimulated lymphocytes (*P* < 0.0001). The CD4^+^FoxP3^+^ Treg cells were significantly induced when stimulated with the CDmix in the presence of IFN-*γ* lymphocytes cocultured with SHED cells (*P* < 0.05). CD4^+^FoxP3^+^ Treg cells were significantly induced when stimulated with CDmix in presence of IFN-*γ* lymphocytes cocultured with DPSCs (*P* < 0.01). CD4^+^FoxP3^+^ Treg cells were significantly induced when lymphocytes stimulated with the CDmix were cocultured with DFSCs (*P* < 0.05). In addition, the CD4^+^FoxP3^+^ Treg cells were significantly induced when stimulated with the CDmix in the presence of IFN-*γ* lymphocytes when cocultured with DFSCs (*P* < 0.05; Figures 8(a) and 8(b)).

### 3.5. Effects of SHEDs, DPSCs, and DFSCs on IL-10, IL-4, and IFN-*γ* Cytokine Expression by Lymphocytes

**Figure 9:**
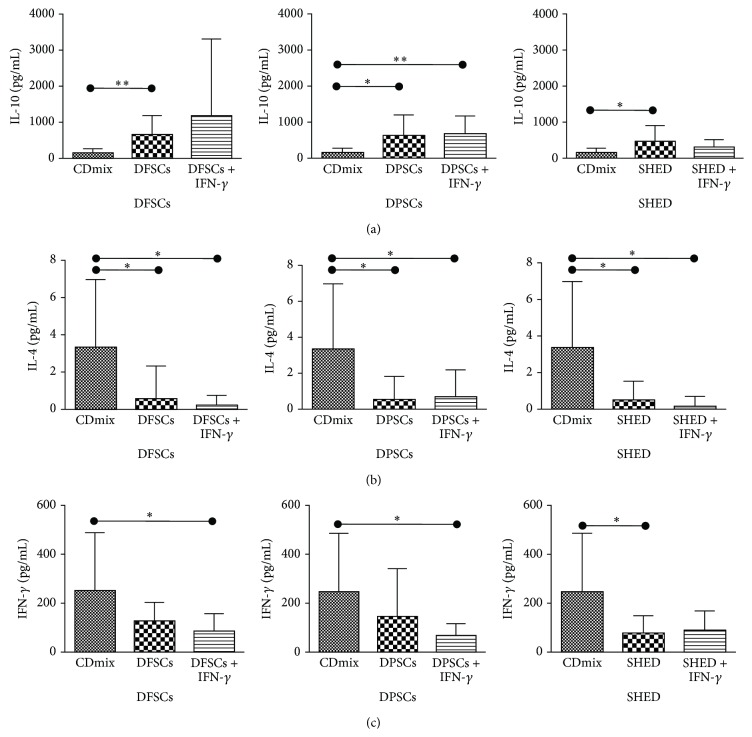
The immunoregulatory effects of SHED, DPSCs, and DFSCs on the expression of IL-10, IL-4, and IFN-*γ* cytokines in lymphocytes. (a) Induced effects of SHED, DPSCs, and DFSCs on the expression of IL-10 cytokines in lymphocytes. (b) Inhibited effects of SHED, DPSCs, and DFSCs on the expression of IL-4 cytokines in lymphocytes. (c) Inhibited effects of SHED, DPSCs, and DFSCs on the expression of IFN-*γ* cytokines in lymphocytes (^*∗*^ *P* < 0.05, ^*∗∗*^ *P* < 0.01).

The expression levels of IL-10, IL-4, and IFN-*γ* were determined via flow cytometry. IL-10 was significantly induced when lymphocytes stimulated with the CDmix were cocultured with the DFSCs (*P* < 0.01) and SHEDs (*P* < 0.05). In addition, IL-10 was significantly induced when lymphocytes stimulated with the CDmix were cocultured with DPSCs (*P* < 0.05) and with the CDmix in the presence of IFN-*γ* when the lymphocytes were cocultured with DPSCs (*P* < 0.01; Figure 9(a)).

IL-4 was significantly inhibited when lymphocytes stimulated with the CDmix were cocultured with DFSCs, DPSCs, and SHEDs (*P* < 0.05). In addition, IL-4 was significantly inhibited when stimulated with the CDmix in the presence of IFN-*γ* when the lymphocytes were cocultured with DFSCs, DPSCs, and SHEDs (*P* < 0.05; Figure 9(b)).

IFN-*γ* was significantly inhibited when lymphocytes stimulated with the CDmix were cocultured with SHEDs (*P* < 0.05). IFN-*γ* was significantly inhibited when stimulated with the CDmix in the presence IFN-*γ* when the lymphocytes were cocultured with DFSCs and DPSCs (*P* < 0.05; Figure 9(c)).

## 4. Discussion

In this study, the immunological impact of SHEDs, DPSCs, and DFSCs was evaluated in vitro. MSCs were first described by Friedenstein in 1968 [7], and the minimal criteria for defining MSCs were developed by the International Society for Cellular Therapy. Accordingly, MSCs must be adherent to plastic surfaces in standard culture conditions and must express CD105, CD73, and CD90, whereas the expression of CD45, CD34, CD14, and CD11b must be absent, and MSCs must be able to differentiate into osteoblasts, adipocytes, and chondroblasts in vitro [23].

MSCs can be isolated from various postnatal regions and tissues, such as cord blood [2, 3], Wharton's jelly [4], the placenta [5, 6], bone marrow [7], teeth [8], and adipose tissue [9, 10]. Dental tissues are promising tissue as a source of MSCs. MSCs in dental tissues have the potential to differentiate into other tissues. Dental tissue can provide dental mesenchymal stem cells, which include SHEDs, DPSCs, DFSCs, apical papilla mesenchymal stem cells (APSCs), and PDLSCs [24, 25]. Dental tissue MSCs represent a source that is easily accessible, and they have the potential to differentiate into other tissue cell lines and can be used to treat several diseases. We have isolated and used three types of dental tissue MSCs: SHEDs, DPSC, and DFSC. Furthermore, we investigated the effects of these MSCs on immune system cells. These cells can also be extracted with minimal invasiveness, unlike other cell types, and are therefore readily accessible.

Miura et al. isolated MSCs from human deciduous teeth; from each deciduous tooth, they obtained 15–20 cells. The authors showed that these cells were adherent to plastic surfaces and had characteristics of stromal cells. When the authors compared these cells to bone marrow stromal cells, they found that human deciduous teeth had a greater potential to proliferate and a higher multiplying potential than the bone marrow stromal cells. In addition, deciduous teeth stem cells expressed STRO-1 and CD146 surface markers [8]. Suchánek et al. isolated SHEDs and showed that these stem cells expressed high levels of CD44, CD73, CD90, CD117, CD166, and HLA I, medium levels of CD29 and CD105, and low levels of CD45, CD63, and CD71 cell surface markers. Additionally, these cells were negative for CD18, CD31, CD34, CD49d, CD49e, CD106, CD133, CD184, CD197, CD146, and HLA II cell surface markers [1]. In our study, we isolated stem cells from human deciduous teeth according to the isolation protocol. These cells were adherent to plastic surfaces and exhibited a high proliferation potential. After isolation, we characterized the cells and showed that they expressed the CD146 surface marker and other stem cell markers, including CD73, CD90, CD105, and CD29, and that they were negative for CD14, CD45, CD34, CD25, and CD28 markers.

Tarle et al. compared the proliferation and differentiation potential and gene expressions of SHEDs and PDLSCs. In their study, they examined the osteogenic, adipogenic, and chondrogenic differentiation of SHEDs and PDLSCs and stained the differentiated cells with Alizarin red, Oil Red O, and von Kossa, respectively, to demonstrate the differentiation process [13]. In our study, we utilized osteogenic, adipogenic, and chondrogenic differentiation protocols and stained the cells with Alizarin red, Oil Red O, and Alcian blue, respectively, showing that SHEDs can differentiate into these three cell lines (osteogenic, adipogenic, and chondrogenic). When osteogenic differentiation was performed, we demonstrated osteoblasts in the cell line. In adipogenic cultures, oil drops were observed, and, in the chondrogenic culture, cartilage and proteoglycans were observed after the staining protocol.

Gronthos et al. isolated MSCs from human dental pulp and found that dental pulp stem cells were inside the mineralized matrix and exhibited fibrosed and blood-veined tissue similar to pulp complexes. Isolated stem cells exhibited morphology of fibroblast-like cell colonies. These cells were compared with bone marrow stem cells and did not express hematopoietic lineage cell surface markers, including CD14, CD34, and CD45 [11]. Another study published in 2010 reported that isolated DPSCs exhibited fibroblast-like colonies and were negative for the CD11b, CD34, CD31, CD33, CD49b, and CD45 cell surface markers and positive for the CD44, CD73, and CD90 cell surface markers [26]. Doğan et al. showed that DPSCs were negative for CD34, CD45, and CD133 and positive for CD29, CD73, CD90, CD105, and CD166 [27]. We isolated dental pulp stem cells and showed that they were confluent on the 9th day in plastic culture flasks. The cells exhibited morphology of fibroblast-like colonies and were positive for CD73, CD90, CD105, CD29, and CD146 and negative for the hematopoietic markers CD14, CD45, CD34, CD25, and CD28.

Doğan et al. isolated DPSCs and performed immunocytochemical analyses and RT-PCR and induced differentiation to osteogenic, adipogenic, and chondrogenic cell lines. The differentiated cells were stained with von Kossa, Oil Red O, and Alcian blue, respectively [27]. Eslaminejad et al. isolated dental pulp stem cells and performed flow cytometry and RT-PCR, assessed the multiplying ratio, and performed odontogenic, chondrogenic, adipogenic, and osteogenic differentiation studies [26]. We isolated DPSCs and performed differentiation studies for osteogenic, adipogenic, and chondrogenic cell lines. After differentiation, we stained the cells with Alizarin red, Oil Red O, and Alcian blue, respectively, to show the differentiation potency. After staining, we examined the cells using a microscope, which revealed osteoblast nodules as osteogenic, oil drops as adipogenic, and proteoglycans as chondrogenic regarding the differentiation processes.

Dental follicle is a surrounding dental tissue that covers the dent. Dental follicles, cementum, periodontal ligaments, and alveolar bone marrow are mesenchymal tissues that together form the dent [28]. Dental follicle is a tissue that is easily accessible. Dental follicles are removed during surgical processes due to orthodontic diseases. Additionally, it is easy to isolate stem cells from dental follicles. Handa and colleagues showed for the first time that DFSCs form a cementum-like matrix when the cells are differentiated in vitro. However, DFSCs form fibroblast-like colonies and are adherent to plastic surfaces [29]. Yokoi et al. showed that DFSCs are able to form periodontal ligaments [28]. Recent studies have also shown that DFSCs have a high proliferation capacity and can be differentiated toward osteogenic, adipogenic, and chondrogenic cell lines [30]. In our study, we isolated stem cells from dental follicles and achieved confluence on plastic surfaces on the 5th day.

Mori et al. isolated DFSCs and performed immunophenotype analyses. These cells expressed the surface markers CD73, CD146, CD90, CD44, CD105, and HLA I and were negative for CD45. Additionally, these cell lines showed a higher proliferation rate compared with bone marrow MSCs [12]. In contrast to the study by Mori et al., we showed that DFSCs do not express hematopoietic stem cell markers (CD14, CD45, CD34, CD25, and CD28). Additionally, DFSCs express CD73, CD90, CD105, CD29, and CD146, which are MSC markers.

Mori et al. showed that DFSCs could differentiate into osteogenic cell lines. We additionally showed that these cells have the potential to differentiate into osteogenic, adipogenic, and chondrogenic cell lines. After the differentiation process, we stained the cell lines with Alizarin red, Oil Red O, and Alcian blue to show the osteogenic, adipogenic, and chondrogenic differentiation processes, respectively. After staining, we examined the cell lines using a microscope and observed that, in the osteogenic cell lines, osteoblasts were stained orange-red indicating osteoblast nodules, oil drops were stained red indicating adipogenic cell lines, and proteoglycans were stained blue indicating chondrogenic cell lines.

We compared three types of MSCs obtained from different dental sources. In addition, we compared the proliferation rate, differentiation potential, and gene expressions from RT-PCR among the DPSCs, SHEDs, and PDLSCs. Comparing the isolation steps, the colony-forming units, and the proliferation potential, the proliferation potential was higher in the DFSCs (DFSC > SHED > DPSC). Additionally, flow cytometry analyses were performed, revealing that the DPSCs expressed higher amounts of MSC markers compared with the SHED cells and DFSCs. The SHEDs expressed lower amounts of hematopoietic stem cell markers compared with DPSCs and DFSCs (SHED > DPSC > DFSC). As a result, DFSCs may be more effective due to their proliferation potential, colony-forming ability, and differentiation potential compared to other cell lines, in particular compared with SHEDs and DPSCs.

Miura and colleagues showed that SHEDs express the ALP gene as a marker of stromal and vascular system cell lines [8]. The ALP gene is a specific marker for nondifferentiated pluripotent stem cells. In addition, induced pluripotent stem cells express ALP [31]. It has also been shown that DFSCs express ALP when cultured in a hypoxic environment [32]. We showed that SHEDs, DPSCs, and DFSCs express the ALP gene. DFSCs expressed higher levels of the ALP gene compared with the SHEDs and DPSCs. In addition, we showed that SHEDs, DPSCs, and DFSCs expressed DSPP, RUNX2, NOTCH, and NESTIN genes. The DFSCs expressed higher levels of DSPP, RUNX2, and NOTCH genes. Compared with SHEDs and DPSCs, the DFSCs expressed higher levels of NANOG, a pluripotent stem cell marker.

Maintaining the continuity of biologic functions is the first target of cell-based therapy for the treatment injured tissues and organs. For this purpose, stem cells are the most important material used in cell-based therapies [33]. Stem cells from various sources (e.g., embryonic stem cells and bone marrow MSCs) have been used in experimental models both in vitro and in vivo. However, ethical considerations impede the use of embryonic stem cells, and the teratoma potential of these cell lines prevents their use in clinical trials [34]. Therefore, stem cell isolation studies now focus on MSCs derived from other tissues, such as muscle, cartilage, dental pulp, adipose tissue, neural tissue, and bone marrow [35].

MSC therapy is a promising biological therapy for the treatment of several diseases. Due to the simplicity of isolating MSCs, their rapid proliferation in culture conditions, and their promising differentiation properties compared to other cell lines, it has been suggested that most MSCs can be used in studies [36, 37]. In addition, MSCs are potential regulators of the immune system, and their teratoma risk is very low [37]. In this study, we examined the effect of MSCs isolated from deciduous teeth, dental pulp, and dental follicle on immune system cells. The results reported here are important for the use of these cell lines for treatment of several immune system diseases.

MSCs are highly promising due to their immunosuppressive and immunomodulatory effects on autoimmune diseases [38, 39]. There are limited experimental studies in this area. MSCs are used to treat diseases such as multiple sclerosis (MS) and amyotrophic lateral sclerosis and are thought to be effective in slowing or inducing regression of these diseases [40]. The immunogenicity of MSCs is very low, and they induce immunosuppressive effects, which represents the primary reason why the researchers have focused on MSCs in clinical applications. These cells do not express HLA-DR or costimulatory factors (CD80, CD86). MSCs stimulate Treg cells and prevent T cell activation, thus inhibiting B cell activation. MSCs present their immunosuppressive effects by suppressing T cell proliferation. In this study, we examined the immunosuppressive effects of SHEDs, DPSCs, and DFSCs by culturing each cell type with immune system cells from peripheral blood mononuclear cells.

MSCs suppress the response of naïve and memory T cells stimulated with mitogens. Demircan and colleagues showed that dental pulp stem cells induce a suppressive effect on T cell proliferation [41]. First, we examined the effects of SHEDs, DPSCs, and DFSCs on lymphocyte proliferation. All groups of MSCs induced suppressive effects on lymphocytes. Additionally, the cultures were separated into two groups: cultures with or without IFN-*γ* stimulation. The DFSCs were the most effective in suppressing lymphocytes in cultures with or without IFN-*γ*. Thus, stem cells exhibit inhibitory effects on T lymphocyte proliferation, in agreement with other studies. For the first time, we found that these cells exhibit the highest immunosuppressive effect in the presence of IFN-*γ*. In addition, the immunosuppressive efficiency of the SHEDs, DPSCs, and DFSCs was compared.

Some previous reports have shown that, after coculture, MSCs suppressed T lymphocytes, but the survival of T lymphocytes increased, and apoptosis decreased [42]. We also showed the suppression of lymphocyte apoptosis. Additionally, we performed a Fas/Fas ligand analysis to study apoptosis. We evaluated the three types of dental MSCs and compared their cocultures with lymphocytes. The results showed that all of the dental MSCs suppressed T lymphocyte apoptosis. In the cocultures with IFN-*γ* stimulation and the DFSCs cultured without IFN-*γ*, the suppression of T lymphocyte apoptosis was significantly decreased. Additionally, when compared to other dental MSCs, cocultures with SHEDs showed that expression of the Fas ligand (CD178) was significantly suppressed with the stimulation of IFN-*γ*.

Demircan and colleagues showed that dental stem cell and T cell proliferation cocultures increase the ratio of CD4^+^ to FoxP3^+^ T cells [41]. Flow cytometric analyses of the cocultures revealed that the numbers of CD4^+^FoxP3^+^ Treg cells increased compared with the lymphocyte cultures with and without MSCs. Additionally, IFN-*γ* stimulation increased the number of CD4^+^FoxP3^+^ Treg cells. The suppression effect of the DFSCs, SHEDs, and DPSCs was also compared. The DFSCs and DFSCs with IFN-*γ* simulation exhibited the highest increase in CD4^+^FoxP3^+^ Treg cells compared with the other dental MSCs.

Demircan and colleagues showed that dental stem cells and T cell proliferation cocultures increase the levels of IFN-*γ* cytokines [41]. Additionally, we analyzed the levels of IL-4 and IL-10 cytokines. After coculturing, the supernatants were collected, and a cytokine analysis was performed. The dental MSCs suppressed the expression of IL-4 and IFN-*γ*, whereas the expression of IL-10 was increased. IL-10 is secreted by macrophages and T lymphocytes and suppresses the expression of IL-12, IFN-*γ*, and TNF-*α* [43, 44].In our study, the MSCs increased the expression of IL-10 and suppressed IFN-*γ*.

## 5. Conclusions

In this study, we examined the immunologic effects of SHEDs, DPSCs, and DFSCs on lymphocytes of healthy donors in vitro. We observed that DFSCs were simply accessible and isolated and were able to differentiate into other cell types more efficiently compared with the other dental MSCs. The DFSCs, SHEDs, and DPSCs suppressed lymphocyte proliferation, increased Treg cells, decreased IL-4 and IFN-*γ* levels, and increased IL-10 levels. Additionally, DFSCs exhibited a higher immunomodulatory effect on immune system cells. Upon stimulation with IFN-*γ*, DFSCs exhibited immunomodulatory functions, suggesting that they can be used for the treatment of autoimmune, inflammatory, and allergic diseases.
